# Case study: Targeted RNA-sequencing of aged formalin-fixed paraffin-embedded samples for understanding chemical mode of action

**DOI:** 10.1016/j.toxrep.2022.04.012

**Published:** 2022-04-18

**Authors:** Michael D. Cannizzo, Charles E. Wood, Susan D. Hester, Leah C. Wehmas

**Affiliations:** a Center for Computational Toxicology and Exposure, Office of Research and Development, US Environmental Protection Agency, Research Triangle Park, NC 27709, USA; b Oak Ridge Institute for Science and Education, US Environmental Protection Agency, Research Triangle Park, NC 27709, USA

**Keywords:** AIC, Akaike Information Criterion, BMD, Benchmark dose, BMD_A_, traditional benchmark dose based on apical endpoint, BMDL, lower 95% confidence limit of benchmark dose, BMDU, upper 95% confidence limit of benchmark dose, BMD_T_, transcriptomic benchmark dose, CPM, counts per million, DCA, dichloroacetic acid, DEG, differentially expressed gene, DOs, detector oligos, FDR, false discovery rate, FFPE, formalin-fixed paraffin-embedded, FROZ, frozen, GO, gene ontology, IRIS, Integrated Risk Information System, NAM, new approach method, Phred score, negative log10 of the base-calling error probability, RIN, RNA integrity number, RNA-Seq, RNA-Sequencing, TempO-Seq, Templated Oligo Sequencing, Formalin-fixed paraffin-embedded (FFPE), RNA-Seq, TempO-Seq, Dichloroacetic acid, DCA, Benchmark dose (BMD) analysis

## Abstract

Formalin-fixed paraffin-embedded (FFPE) samples are the only remaining biological archive for many toxicological and clinical studies, yet their use in genomics has been limited due to nucleic acid damage from formalin fixation. Older FFPE samples with highly degraded RNA pose a particularly difficult technical challenge. Probe-based targeted sequencing technologies show promise in addressing this issue but have not been directly compared to standard whole-genome RNA-Sequencing (RNA-Seq) methods. In this study, we evaluated dose-dependent transcriptional changes from paired frozen (FROZ) and FFPE liver samples stored for over 20 years using targeted resequencing (TempO-Seq) and whole-genome RNA-Seq methods. Samples were originally collected from male mice exposed to a reference chemical (dichloroacetic acid, DCA) at 0, 198, 313, and 427 mg/kg-day (n = 6/dose) by drinking water for 6 days. TempO-Seq showed high overlap in differentially expressed genes (DEGs) between matched FFPE and FROZ samples and high concordance in fold-change values across the two highest dose levels of DCA *vs.* control (R^2^ ≥ 0.94). Similarly, high concordance in fold-change values was observed between TempO-Seq FFPE and RNA-Seq FROZ results (R^2^ ≥ 0.92). In contrast, RNA-Seq FFPE samples showed few overlapping DEGs compared to FROZ RNA-Seq (≤5 for all dose groups). Modeling of DCA-dependent changes in gene sets identified benchmark doses from TempO-Seq FROZ and FFPE samples within 1.4-fold of RNA-Seq FROZ samples (93.9 mg/kg-d), whereas RNA-Seq FFPE samples were 3.3-fold higher (310.3 mg/kg-d). This work demonstrates that targeted sequencing may provide a more robust method for quantifying gene expression profiles from aged archival FFPE samples.

## Highlights

• Targeted RNA-Seq outperformed traditional RNA-Seq DCA changes in gene expression of >20 yr old FFPE liver samples.
• Targeted RNA-Seq of FFPE showed improved gene expression concordance and BMD estimates with paired FROZ samples as opposed to RNA-Seq of FFPE.
• Exon-targeted probes and direct lysis of FFPE samples for targeted RNA-Seq may circumvent some of the fixation-related damage to nucleic acids.
• Gene set-based BMD estimates from short-term studies may be useful in measuring biologic potency of chemicals in the absence of long-term studies.

## Graphical Abstract

**Figure:**
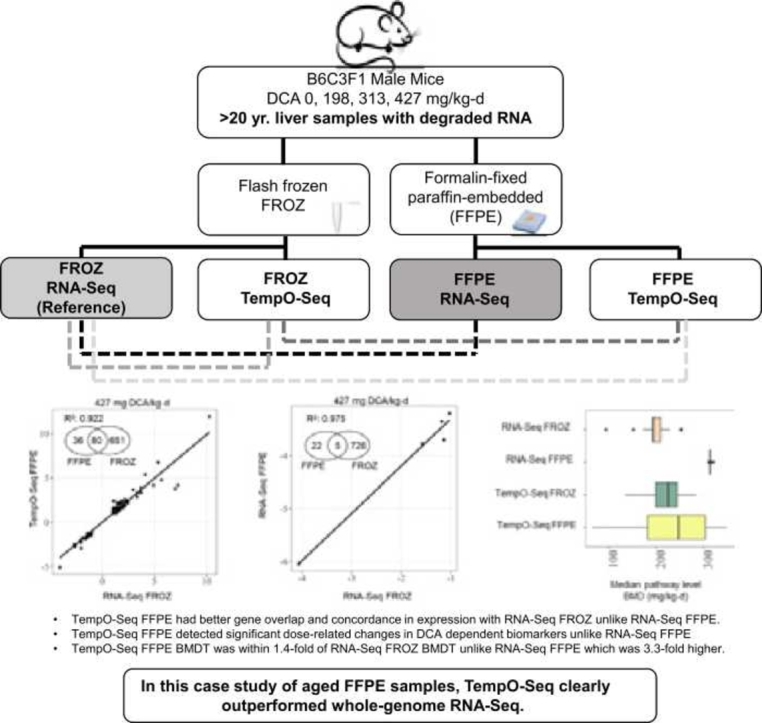

## 1 Introduction

In recent years there has been increasing focus on the use of alternative or new approach methods (NAMs) for human health and safety assessment of chemicals. These methods are intended to increase efficiency and human relevance of toxicity testing while reducing reliance on traditional animal studies [24], [30]; [13]. A cornerstone of these efforts is the identification of molecular biomarkers that can be anchored to target pathways and ultimately used to predict adverse phenotypic effects. Biorepositories, which contain millions of curated tissue samples, often from unique toxicological and clinical studies, provide a rich and ready-made resource for biomarker discovery, adverse outcome pathway development, and dose response analysis. Greater availability of molecular data from archival samples directly linked to a pathologic outcome would support NAM efforts and enable more rapid chemical risk prioritization, while reducing the need for new *in vivo* studies.

Benchmark dose (BMD) analysis has been used for several decades to determine chemical potency and health guidance values based on modeling of adverse phenotypic responses. More recently, multiple studies have shown that BMD analysis of gene expression data from short term *in vivo* studies can provide useful surrogates for traditional morphologic or “apical” endpoint BMDs [12], [16], [18], [19], [33], [4]. Archival tissue samples may provide a convenient and large-scale resource for this latter approach, by anchoring different NAMs to adverse phenotypic effects *via* short term gene expression responses. Historically, formalin fixation-induced changes in archival samples have been a major obstacle to this approach, given that the vast majority of samples within biorepositories are stored as formalin-fixed paraffin-embedded (FFPE) blocks. While this preservation method works well for long term preservation of tissue morphology, it introduces damage to nucleic acids and other macromolecules in fixed tissues over time which limits their use for gene expression analyses [15], [18], [28], [36].

Advances in RNA-Sequencing technologies (RNA-Seq) are helping to overcome the challenges associated with obtaining reliable gene response data from FFPE samples. One of the major factors driving lower quality RNA from FFPE samples is age in FFPE block, which increases RNA fragmentation and causes chemical modifications like formaldehyde adducts and covalent crosslinks [11], [2], [22], [23], [25]. While specialized RNA extraction techniques [10], [22], [28], [39], ribo-depletion RNA-Seq library preparations [37], and whole-genome RNA-Seq methods [18], [26], [27] can help rescue gene expression signals from recently archived FFPE samples, these approaches have shown limited success for aged samples stored for extended periods of time (>10 years). In one example [18], RNA-Seq analysis of more recently collected FFPE samples (~2 years old) showed highly concordant gene responses compared to fresh-frozen. In contrast, the same methods applied to aged FFPE tissue samples (>20 years old) detected only the most abundant transcripts and showed dramatic reductions in transcript alignments and marker gene counts (−88% and −97%, respectively) relative to their fresh-frozen pairs, highlighting the deleterious impact of age in FFPE block [18].

More recently, several studies have described a probe-based technique called Template Oligo Sequencing, or TempO-Seq, for gene expression profiling without the need for RNA isolation or pre-amplification [29], [41], [5]. Notably, this method was able to detect consistent gene expression profiles from decades-old FFPE cancer tissue replicates from the same animal or human donor [34] and demonstrate concordant gene expression data between flash-frozen (FROZ) and FFPE samples 5–10 years old [35]. Compared to standard whole-genome RNA-Seq methods, TempO-Seq uses direct detector oligo (DO) hybridization rather than reverse transcription. DOs anneal to RNA sequences of < 100 nt potentially making this platform better suited to samples with highly degraded RNA.

**Fig. 1:**
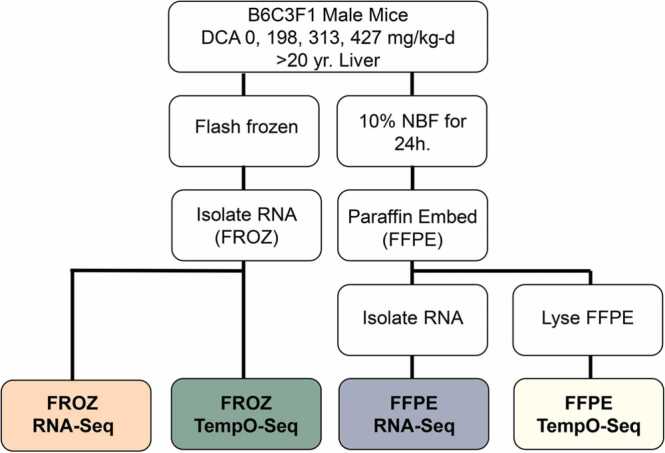
Experimental overview. The goal of the study was to evaluate gene expression profiles using TempO-Seq on aged formalin-fixed paraffin-embedded (FFPE) samples compared to paired frozen (FROZ) samples (n = 6/dose level/preservation). Previous RNA-Sequencing (RNA-Seq) profiles were used as referent data (n = 6/dose level/preservation). Samples consisted of paired liver tissue from male mice exposed to multiple dose levels of dichloroacetic acid (DCA) by drinking water for 6 days that were either flash frozen or formalin-fixed in 10% neutral buffered formalin (NBF) for 24 h. followed by paraffin-embedding. Samples were then subjected to RNA-Seq and TempO-Seq > 20 years later.

The goal of this case study was to evaluate the quality metrics and quantitative dose responses in gene expression data from aged FROZ and FFPE tissue sample pairs (>20 years old) using the TempO-Seq platform and to compare these profiles with those obtained using Illumina whole-genome RNA-Seq technology. To do this, we identified differentially expressed genes (DEGs) induced by a well-studied reference chemical (dichloroacetic acid, DCA) in paired FFPE and flash-frozen (FROZ) samples using TempO-Seq and then compared those results to previous total RNA-Seq data generated from the same paired samples (Fig. 1). We should note that although many biorepositories contain older FFPE samples, it is exceedingly rare to have paired FROZ and FFPE tissue samples > 20 years old from a controlled multi-dose chemical toxicology study. This sample set thus provided a unique opportunity to evaluate sequencing methods specifically in aged samples. As a proof-of-concept application, we also quantified dose-dependent changes in coordinated gene responses across these two platforms and evaluated BMD estimates for transcriptional *vs.* apical effects.

## 2 Materials and methods

### 2.1 Study design

Paired archival FFPE and FROZ liver samples were collected from 4-week-old male B6C3F1 mice in a 6-day cell proliferation experiment completed in 1994. DCA (CAS 79–43–6, Aldrich, Milwaukee, WI) was administered to mice *via* drinking water at 0, 1.0, 2.0 and 3.5 g/L (as described in DeAngelo et al. [42]). Daily intake values were estimated at 0, 198, 313 and 427 mg/kg-d [18]. All animals were maintained under standard housing conditions at the U.S. Environmental Protection Agency (U.S. EPA) AAALAC-accredited facility (Research Triangle Park, NC). Animal care and use protocols were approved by the Institutional Animal Care and Use Committee at U.S. EPA.

At necropsy, each liver sample was divided and either flash frozen (FROZ) in liquid nitrogen before storage at − 70 to − 80 °C (U.S. EPA in Research Triangle Park, NC) or fixed in commercial 10% buffered formalin for ~24 h before transfer to 70% ethanol as described in Hester et al. [18]. Formalin fixed tissue samples then underwent standard histology processing, which occurred within 18 months of sample collection. After processing, FFPE samples were stored at ambient temperature in a warehouse (U.S. EPA Archives, Arlington, VA). FROZ samples were collected from the left lateral caudate and right medial liver lobes, whereas FFPE samples were collected from the left lateral and right medial liver lobes. RNA isolation and RNA-Seq occurred ~21 years after collection and preservation.

### 2.2 RNA isolation, sample preparation and sequencing

#### 2.2.1 RNA sequencing

RNA was isolated from aged FROZ and FFPE samples (n = 6 per dose group for each preservation), as previously described in Lake et al. [43] and Hester et al. [18], respectively. Briefly, RNA from FROZ samples was isolated using homogenization and RNAzol® RT (cat #RN 190; Molecular Research Center, Cincinnati, OH) followed by purification using a RNeasy MinElute column (Qiagen, GmbH, Hilden, Germany). RNA purity and concentration were assessed by Agilent Bioanalyzer (RNA 6000 Pico Kit cat #5067–1514 or Nano Kit 5067–1529; Agilent Technologies GmbH, Berlin, Germany), NanoDrop™− 2000 spectrophotometer (ThermoFisher Scientific, Waltham, MA), and Qubit fluorometer (RNA BR Assay Kit cat #Q10210 or HS Assay Kit cat #Q32852; ThermoFisher Scientific, Waltham, MA).

FFPE RNA for total RNA-Seq was isolated using 2, 10-µm sections that were deparaffinized (Deparaffinization Solution, cat #19093; Qiagen), protease-digested at 56 °C for 15 min, incubated at 80 °C for 15 min, DNAse-treated, and then purified on RNeasy MinElute spin column (Qiagen AllPrep® DNA/RNA FFPE Kit; cat #80234) according the manufacturer protocol. RNA concentration and purity were assessed the same way for FFPE and FROZ samples.

RNA-Seq data from FROZ and FFPE samples were previously generated and analyzed as described in Hester et al. [18]. The FASTQ files are available through Gene Expression Omnibus (www.ncbi.nim.nih.gov/geo/; GEO Accession #GSE78962). Briefly, RNA was converted to cDNA using Illumina TruSeq Stranded Total RNA preparation kit (#RS-122–2303, Illumina, San Diego, CA). Ribosomal RNA was removed by a biotinylated probe (Ribo-Zero Gold Library Prep Kit, #RS-122–2303, Illumina, San Diego, CA). Samples were then purified and fragmented *via* incubation with divalent cations. Fragmentation times were reduced for FFPE samples. Size distribution and integrity were measured using an Agilent Bioanalyzer (DNA 1000 kit #5067–1504). cDNA libraries were quantitated by qPCR (KAPA Library Quant Kit #KK4824, KAPA Biosystems, Wilmington, MA) and normalized to 2 nM. Samples were labeled with barcodes, pooled, and sequenced to a target read depth of 25 million at Expression Analysis (EA Genomic Services, Q2 Solutions -- a Quintiles Quest Joint Venture, Durham, NC) with 8 samples per sequencing lane using the Illumina HiSeq 2000 platform with 2x50bp-paired end reads. The mean RNA integrity number (RIN) values for FROZ and FFPE samples were 3.09 and 2.41 respectively with standard deviations of 0.49 and 0.50. The mean RNA 260/280 values for FROZ and FFPE samples were 1.72 and 1.88 respectively with standard deviations of 0.05 and 0.04.

#### 2.2.2 TempO-seq

Previously isolated FROZ RNA was used in the TempO-Seq assay. FFPE samples used 2 adjacent, 10-μm slices of the same FFPE liver samples (now ~24 years-old) used for total RNA-Seq. FFPE sections were treated with mineral oil to remove paraffin and then lysed using BioSpyder Lysis buffer. Libraries were prepared as described in Trejo et al. [34]. FROZ RNA or FFPE lysate was processed in annealing buffer with DOs, probes that have high selectivity for 21,451 *Mus musculus* target genes (Whole Genome Mouse Assay, BioSpyder Technologies; Carlsbad, CA). After oligo ligation, a nuclease mixture was added to digest unbound or erroneously bound DOs. A final ligase mix was added to facilitate addition of a single primer pair. The ligases were deactivated with heat and the probes were amplified by PCR, which added sample specific barcodes that flank the target sequence and are inserted into the standard Illumina adaptors. PCR-amplified and barcoded samples were then pooled into a single library, purified, and sequenced. TempO-Seq RNA samples were sequenced on the Illumina HiSeq 2500 platform with 50-bp single-end reads to a target read depth of 5 million. Overly abundant transcripts were attenuated equally across all samples to save sequencing reads. The adjustment factors are reported in (Supplementary Table 1). Assay controls identified 95.7% mapped reads (>7.78 million reads) in the positive control with a signal to noise ratio of 303:1. TempO-Seq FASTQ files are available from NCBI GEO accession #GSE186113.

#### 2.2.3 Sequencing analyses

Sequenced data were analyzed using Partek Flow (Partek build version 9.0.20.0202, St. Louis, MO). Prior to upload into Partek Flow, total RNA-Seq FASTQ files were demultiplexed and clipped using EA-utils tools package to remove sequencing adapters and low-quality bases from both ends of reads (EA Genomic Services, Q2 Solutions -- a Quintiles Quest Joint Venture, Durham, NC). Poor quality sequences were filtered if they had an average Phred score < 25, consisted of homopolymers, or were < 25 nucleotides long. TempO-Seq sequencing reads were demultiplexed and adapters trimmed using standard Illumina sequencer software to generate FASTQ files for each sample. Both datasets were uploaded to Partek Flow for alignment and differential expression analysis. Paired-end RNA-Seq reads and single-end TempO-Seq reads were each aligned to *Mus musculus* transcriptome (MM10) using Spliced Transcripts Alignment to a Reference (STAR, v2.6.1d). Aligned total RNA-Seq reads and TempO-Seq reads were quantified into gene counts using Partek Expectation Maximization and RefSeq Transcripts 92 annotation build. A zero-count offset of 0.0001 was added to all genes across all samples prior to filtering. This offset is recommended for the Partek analysis pipeline. Genes with low expression (geometric mean ≤ 1 across) across all samples regardless of treatment status were removed. Counts were CPM-normalized (count per million mapped reads to adjust for read differences between sample libraries), log2 transformed, and analyzed for significant differentially expressed genes (DEGs) using Partek Gene Specific Analysis (GSA) to multiple models (negative binomial, normal and log-normal). The lowest corrected Akaike Information Criterion (AIC) was used to select the gene model fit. Significant DEGs were defined as genes with false discovery rate (FDR)-adjusted p-value < 0.05 [3] and absolute fold change > 2.

#### 2.2.4 Benchmark dose modeling

BMD analysis identifies the dose at which a traditional apical endpoint like liver weight or a nontraditional endpoint like gene expression change compared to vehicle controls by a set benchmark response factor (BMR) such as 10%. For this study, DCA concentrations were converted to estimated intake values of 0, 198, 313 and 427 mg/kg-day [18]. The apical BMD calculated for the U.S. EPA Integrated Risk Information System (IRIS) risk assessment of DCA (EPA 2003) used data from DeAngelo et al. [44]. Data were converted to human equivalent dose in the IRIS document. Therefore, to be comparable with mouse, we employed U.S. EPA Benchmark Dose Software (BMDS, v3.2) to calculate the mouse BMD using a BMR= 0.10 and settings recommended in the Benchmark Dose Technical Guidance Document [9]. As with the IRIS assessment, the high dose level was excluded from the analysis, the incidence of hepatocellular adenomas or carcinomas was fit, and the model with the best fit p-value and lowest AIC was used to set the BMD. The full results are included in Supplementary Table 2.

For the gene expression data, low count filtered and CPM-normalized FROZ and FFPE count matrices were loaded into BMDExpress v2.3 (https://github.com/auerbachs/BMDExpress-2/releases; [31]) and analyzed separately. The only difference with these count matrices compared to those used for Partek Flow is that a zero-count offset of 1 was used prior to log_2_ transformation. BiomaRt [6], [7] was used to convert gene symbols to Ensembl identifiers for BMD analysis and gene identification mapping in R v4.0. BMDExpress analysis proceeded as follows. Genes were initially filtered by ANOVA (p-value <0.05) and a maximum absolute fold change > 2. Data were then fitted assuming constant variance to linear, power, Hill model, polynomial (2 and 3) and exponential (2, 3, 4 and 5) models with a BMR set at 1.349 times the standard deviation of the vehicle controls with a 10% increase in tail area [40]. The best polynomial model was selected based on a Nested Chi Square Test, and best overall model was selected based on the lowest AIC. The Hill model was flagged if the ‘k’ parameter was < 1/3 the lowest positive dose. In the case where a flagged Hill model was identified as best overall model fit, the next best model with a p-value > 0.05 was selected. Genes were mapped to Reactome pathways and gene ontology (GO) biological process terms for identification of gene set BMDs in BMDExpress v2.3. Enrichment required at least 3 genes to map, a ratio between the upper and lower 95% confidence limit of the BMD (BMDU:BMDL ratio) < 40, a BMD < highest tested dose and a Two-Tailed Fisher’s Exact Test, p-value < 0.05. The lowest median pathway or GO term was used to set the final gene set or transcriptomic BMD (BMD_T_). If there were multiple pathways tied for the lowest median pathway or GO term BMD_T_ and BMDL_T_, the pathway with the lowest Two-Tailed Fisher’s Exact Test was used. This approach was selected as it has previously been shown to provide BMD_T_ estimates that tend to be conservative if not consistent, within 3-fold, of the traditional apical BMDs, when focusing on target tissues [33]. The BMDExpress outputs from ANOVA filtering, model fits, and mapping to enriched gene sets can be found in Supplementary Tables 3, 4 and 5, respectively.

#### 2.2.5 Other statistical analyses

The influence of preservation (FROZ *vs.* FFPE) on sequencing post-alignment metrics and biomarker genes was assessed for normality and homogeneity of variance using the Shapiro Wilk Test and Levene’s Test, respectively. Most of these data were not normally distributed, and the sample size was relatively small; thus, the Wilcoxon Signed Rank Test for nonparametric, paired data was used to determine whether the FFPE groups were statistically different from matched FROZ controls in R v4.0 [32] using the stats, car and clinfun packages. For determining dose-dependent DCA effects of marker genes by platform and preservation method, we first employed a Kruskal-Wallis Rank Sum Test to determine group level differences (DCA *vs.* control) and then a Jonckheere Trend Test (two-sided) to see if there were any dose-dependent effects. If significant group level and dose-dependent differences were observed, we employed a Wilcoxon Rank Sum Test (two-sided) with a Benjamini-Hochberg FDR correction for multiple comparisons (corrected p-value <0.05) [3]. Summary statistics are reported as mean ± standard deviation and significance determined by p-value < 0.05 unless otherwise indicated.

## 3 Results

### 3.1 Global alignment metrics reflect platform dependent differences

We used a paired study design with matching FROZ and FFPE liver tissue samples from DCA-exposed mice to evaluate TempO-Seq relative to established RNA-Seq technology (Fig. 1). Overall, we observed relatively good RNA quality of the > 20 yr. old FROZ samples compared to the matched FFPE samples. This was not immediately obvious when comparing RIN values (<4) but was apparent from global alignment results. RNA-Seq FFPE detected between 26.6 and 33.7 million reads with very low percent alignment (range: 5.0–14.1%) to the reference genome across the different dose levels compared to FROZ, which detected 31.0–32.8 million reads and 87.7–88.5% alignment (Supplementary Table 6). Most RNA-Seq FFPE quality metrics displayed unfavorable differences with matched RNA-Seq FROZ, including significantly reduced percent read coverage of the reference assembly (by 88.7–98.3%, Wilcoxon Signed Rank test, p-value <0.05) and significantly reduced percent unique read alignment (by 83.0–93.8%) (Supplementary Table 6). RNA-Seq FFPE also demonstrated dramatic shifts in location of alignment compared to matched FROZ. On average, RNA-Seq FFPE had 77.2–82.3% alignments to either intron and intergenic regions, or non-protein coding potions of the genome, while matched FROZ had 42.0–44.3%. Only 16.3–21.8% of RNA-Seq FFPE samples reads mapped to exonic regions, whereas the percent of RNA-Seq FROZ reads mapped to exon regions was significantly higher ranging from 46.7% to 48.3% (Fig. 8) RNA-Seq FFPE samples also demonstrated 97.9–99.7% fewer exon to exon junctions compatible with the reference assembly compared to RNA-Seq FROZ (Supplementary Table 7).

**Fig. 2:**
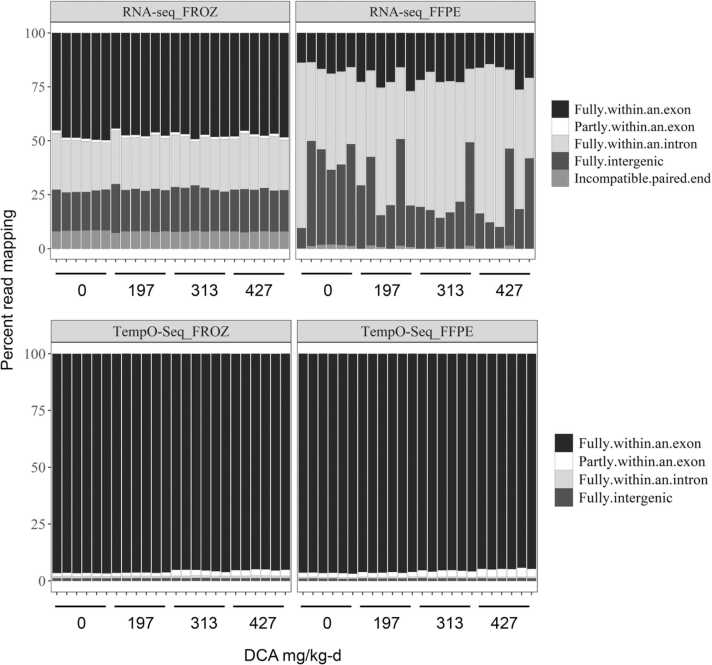
Read alignment across TempO-Seq and RNA-Seq FFPE and matched FROZ samples. RNA-Seq method used paired end reads which requires both ends to align within a region to be considered fully exonic, intronic, or intergenic. TempO-Seq uses a single read. FFPE-formalin-fixed paraffin-embedded, FROZ-frozen, seq-sequencing.

Conversely, TempO-Seq had fewer total reads mapped for FROZ and FFPE (between 8.3 and 9.5 and 7.7–8.4 million reads, respectively) compared to RNA-Seq. This is at least partly due to the read attenuation step that occurs during the TempO-Seq method. Unlike RNA-Seq, the difference between total mapped reads for TempO-Seq FFPE was not always significantly less than TempO-Seq FROZ, depending upon the dose level (Supplementary Table 6). Both paired TempO-Seq sample types also maintained a higher proportion of alignment to the reference genome at 98.1–98.5% for FROZ and 50.1–71.2% for FFPE. While TempO-Seq FFPE samples had several significant differences in quality metrics compared to paired TempO-Seq FROZ, the magnitude of difference was less drastic than that for RNA-Seq results. TempO-Seq FFPE had reduced read coverage (by 27.2–39.0%) and reduced unique read alignment (by 26.4–49.2%) compared to TempO-Seq FROZ but nowhere near as high as the differences observed between RNA-Seq FROZ and FFPE. Platform differences in alignment specificity could partially explain the less drastic preservation-related differences in quality metrics for TempO-Seq samples. For instance, both FROZ and FFPE TempO-Seq samples consistently had > 94% of reads aligned to exon regions. Because TempO-Seq probes exclusively target exon regions, intronic and intergenic read alignments remained low at < 4%. Furthermore, TempO-Seq FFPE had around 5 times the mean proportion of exon-aligned reads compared to RNA-Seq FFPE (Fig. 2). Detection of compatible exon to exon junctions was significantly reduced in TempO-Seq FFPE relative to TempO-Seq FROZ (by 59 ± 4.6%) but not as extreme as the decline observed for RNA-Seq FFPE (Supplementary Table 7).

### 3.2 Gene responses were concordant between paired samples and across technologies

**Fig. 3:**
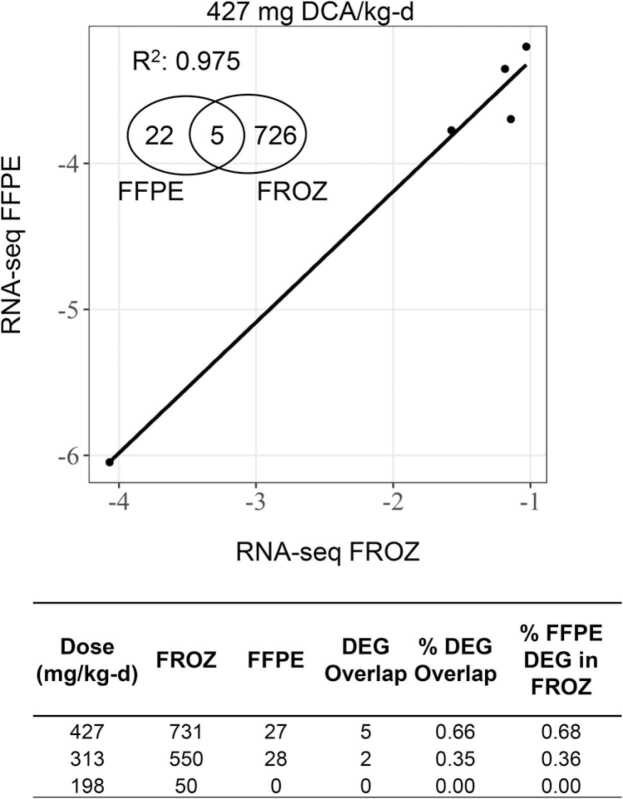
Comparison of RNA-Seq DEGs induced by DCA between paired FROZ and FFPE liver tissue samples reveal few DEGs in FFPE samples across all dose levels. A. Regression analysis of the intersection in log_2_-transformed fold-change values in DEGs between paired RNA-Seq FFPE and RNA-Seq FROZ liver tissue samples induced by DCA at 427 mg/kg-d *vs.* vehicle control B. Total RNA-Seq DEGs induced by DCA in paired FROZ and FFPE tissue samples and overlap in DEGs relative to FROZ samples. DEGs were defined with these parameters: absolute value (fold change)> 2, FDR-adjusted p-value< 0.05. Percent DEG overlap calculated as DEG Overlap/(FFPE + FROZ DEGs)* 100. Percent FFPE DEG in FROZ calculated as (DEG Overlap/FROZ DEGs)* 100. Abbreviations. DEG-differentially expressed genes, DCA- dichloroacetic acid, FFPE-formalin-fixed paraffin-embedded, FROZ-frozen, RNA-Seq- RNA-Sequencing, FDR- false discovery rate.

RNA-Seq of FROZ samples detected a dose-dependent increase in the number of DEGs from 50 to 731 (for 198–427 mg DCA/kg/d) compared to vehicle controls, whereas paired FFPE samples had drastically reduced detection of DEGs (0−28) across all dose levels. The low number of FFPE DEGs showed minimal overlap with paired FROZ (only 2 and 5 DEGs at the two highest DCA dose levels). Despite a large loss in gene counts, RNA-Seq FFPE fold changes across DEGs were still highly concordant with paired FROZ at the 427 mg/kg-d dose (R^2^ = 0.975; n = 5 common genes), suggesting that the detectable changes for the RNA-Seq FFPE groups were due to DCA treatment and not noise (Fig. 3). When RNA-Seq FROZ DEGs were compared to common genes that did not meet statistical thresholds detected in paired FFPE samples, a few additional common genes were identified between groups (3 and 6 genes for 313 and 427 mg/kg-d doses, respectively).

**Fig. 4:**
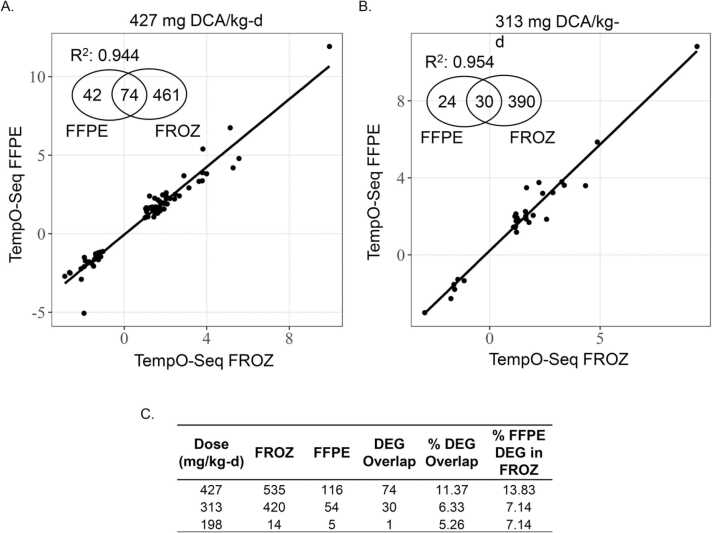
TempO-Seq identified more DCA-induced DEGs than RNA-Seq in aged FFPE liver samples and showed better concordance with paired FROZ samples. Regression analysis of the intersection in log_2_-transformed fold-change values in DEGs between paired TempO-Seq FFPE and TempO-Seq FROZ liver tissue samples induced by DCA at A. 427 mg/kg-d *vs.* vehicle control and B. 313 mg/kg-d *vs.* vehicle control. C. Total DCA-induced DEGs from paired TempO-Seq FROZ and FFPE tissue samples and overlap in DEGs relative to FROZ samples. DEGs were defined with these parameters: absolute value (fold change)> 2, FDR-adjusted p-value< 0.05. Percent DEG overlap calculated as DEG Overlap/(FFPE + FROZ DEGs)* 100. Percent FFPE DEG in FROZ calculated as (DEG Overlap/FROZ DEGs)* 100. Abbreviations. DEG-differentially expressed genes, DCA- dichloroacetic acid, FFPE-formalin-fixed paraffin-embedded, FROZ-frozen, RNA-Seq- RNA-Sequencing, FDR- false discovery rate.

As with RNA-Seq FROZ tissue samples, TempO-Seq FROZ samples demonstrated a dose-dependent increase in DEGs ranging from 14 for the 198 mg/kg-d dose to 535 for the 427 mg/kg-d dose compared to vehicle controls. A similar trend was also observed in the paired TempO-Seq FFPE samples, which had 5–116 DEGs across dose groups and showed more DEGs at every dose compared to RNA-Seq FFPE. When comparing common DEGs between FFPE and matched FROZ, TempO-Seq had a higher number of DEG overlap with increasing dose (1–74 DEGs). There was also high agreement in fold changes between paired TempO-Seq FROZ and FFPE samples among common DEGs for the top two dose levels (R^2^ = 0.954 and 0.944 for 313 and 427 mg/k-d, respectively) (Fig. 4). This concordance remained relatively high for comparison with any TempO-Seq FFPE gene that matched TempO-Seq FROZ DEGs by dose level (R^2^ = 0.779 – 0.828), whereas overlapping genes numbers increased from 9 to 303 across each dose level (Supplementary Figure 1).

**Fig. 5:**
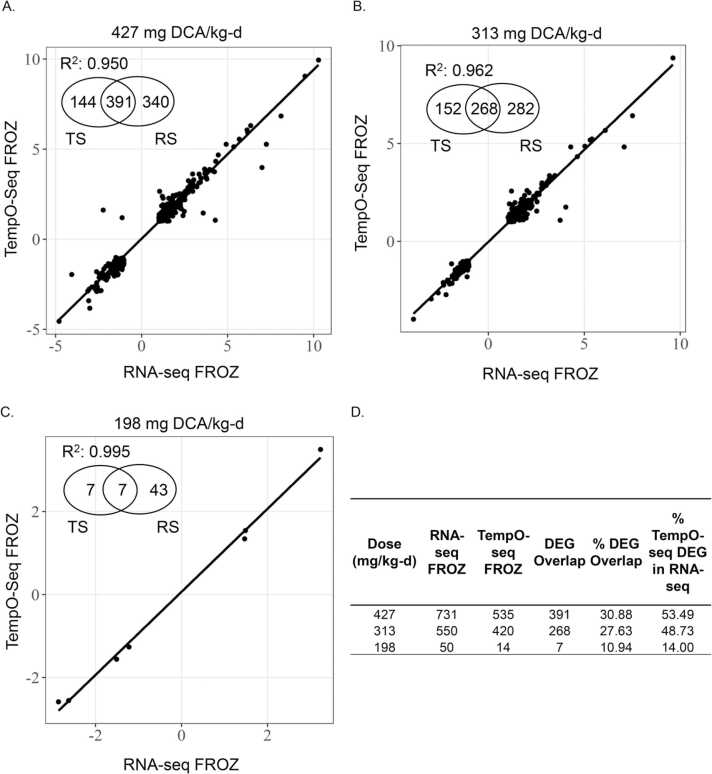
DCA-induced DEGs detected by TempO-seq in FROZ liver tissue remarkably consistent with DEGs detected by RNA-Seq of FROZ liver tissue. Regression analysis of the intersection in log_2_-transformed fold-change values in DEGs between TempO-Seq FROZ and RNA-Seq FROZ liver tissue samples induced by DCA at A. 427 mg/kg-d *vs.* vehicle control, B. 313 mg/kg-d *vs.* vehicle control and C. 198 mg/kg-d *vs.* control D. Total DCA-induced DEGs from TempO-Seq FROZ and RNA-Seq FROZ liver tissue samples as well as overlap in DEGs relative to RNA-Seq FROZ. DEGs were defined with these parameters: absolute value (fold change)> 2, FDR-adjusted p-value< 0.05. DEG overlap includes DEGs in common between RNA-Seq and TempO-seq. Percent DEG overlap calculated as DEG Overlap/(RNA-Seq + TempO-seq DEGs)* 100. Percent TempO-seq DEG in RNA-Seq calculated as (DEG Overlap/RNA-Seq DEGs)* 100. Abbreviations. DEG-differentially expressed genes, DCA- dichloroacetic acid, FFPE-formalin-fixed paraffin-embedded, FROZ-frozen, RNA-Seq- RNA-Sequencing, FDR- false discovery rate.

**Fig. 6:**
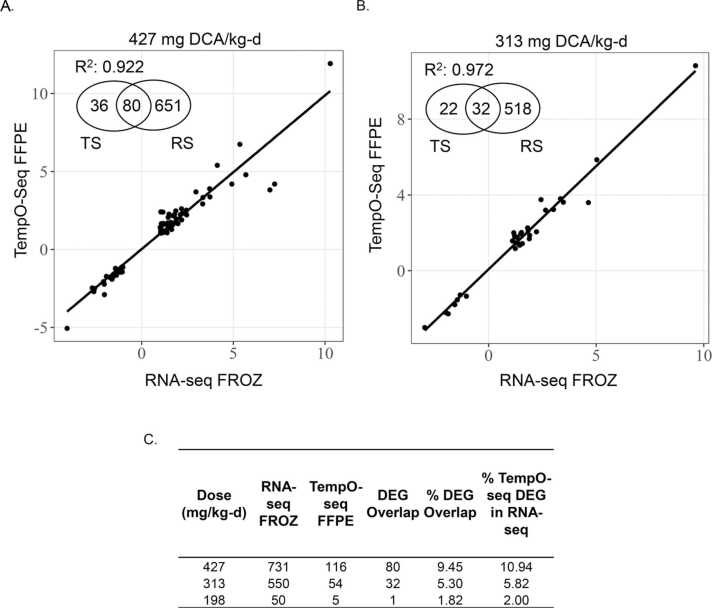
DCA-induced DEGs detected by TempO-Seq in FFPE liver tissue more consistent with DEGs detected by RNA-Seq of FROZ liver tissue than RNA-Seq of FFPE. Regression analysis of the intersection in log_2_ transformed fold-change values in DEGs between TempO-Seq FFPE and RNA-Seq FROZ liver tissue samples induced by DCA at A. 427 mg/kg-d *vs.* vehicle control and B. 313 mg/kg-d. *vs.* vehicle control C. Total DCA-induced DEGs from TempO-Seq FFPE and RNA-Seq FROZ liver tissue samples as well as overlap in DEGs relative to RNA-Seq FROZ. DEGs were defined with these parameters: absolute value (fold change)> 2, FDR-adjusted p-value< 0.05. DEG overlap includes DEGs in common between RNA-Seq and TempO-seq. Percent DEG overlap calculated as DEG Overlap/(RNA-Seq + TempO-seq DEGs)* 100. Percent TempO-seq DEG in RNA-Seq calculated as (DEG Overlap/RNA-Seq DEGs)* 100. Abbreviations. DEG-differentially expressed genes, DCA-dichloroacetic acid, FFPE-formalin-fixed paraffin-embedded, FROZ-frozen, RNA-Seq- RNA-Sequencing, FDR- false discovery rate.

Agreement in gene expression data was also seen across platforms. RNA-Seq FROZ and TempO-Seq FROZ not only identified similar numbers of DEGs across DCA dose levels but also good overlap in DEGs relative RNA-Seq FROZ (7–391 DEGs) with consistent fold changes in gene expression across platforms. The higher number of DEGs in RNA-Seq FROZ was expected, given that the method is not limited to a finite number of probes like the TempO-Seq platform. Despite this, there were still 391 DEGs shared between RNA-Seq and TempO-Seq FROZ at the 427 mg/kg-d dose, which was higher than the 144 TempO-Seq FROZ and 340 RNA-Seq FROZ unshared DEGs. Fold changes were also highly correlated (R^2^ = 0.950). This correlation in expression extended to the other dose levels, with R^2^ = 0.995 and 0.962 for 198 and 313 mg/kg-d dose groups, respectively (Fig. 5). Due to the overall loss in gene counts for RNA-Seq FFPE samples, comparisons to TempO-Seq (FROZ or FFPE) were not very informative. No more than 5 DEGs were shared with any TempO-Seq DEGs (Supplementary Table 8). However, when comparing the DEGs identified from TempO-Seq FFPE samples to RNA-Seq FROZ DEGs, we identified up to 80 DEGs per dose group, which showed highly concordant fold changes at the 313 and 427 mg/kg-d dose levels (R^2^ = 0.972 and 0.922, respectively; Fig. 6). If the comparison was expanded to include any genes present in TempO-Seq FFPE that matched RNA-Seq FROZ DEGs at each dose level, the range in common genes increased from 14 at the 198 mg/kg-d dose to 308 at the 427 mg/kg-d dose. The concordance in fold-change values was slightly less than TempO-Seq FROZ *vs.* RNA-Seq FROZ but still high (R^2^ =0.767 – 0.902; Supplementary Figure 2).

### 3.3 Marker gene response shows TempO-Seq FFPE is more sensitive than Total RNA-Seq FFPE

We next investigated marker gene families present in both platforms that were known or likely to be influenced by DCA exposure (*i.e.* Acyl-CoA thioesterases (*Acot*) and Cytochrome p450 (*Cyp*) families) [18]. Here, RNA-Seq of FROZ samples detected 11 *Acot* and 43 *Cyp* genes with significant dose-dependent trends (Kruskal-Wallis test, p-value <0.05 and Jonckheere’s trend test, p-value <0.05). For paired FFPE, RNA-Seq identified only 2 *Acot* and 24 *Cyp* genes with significant dose-dependent trends in gene expression. Of these, 2 *Acot* and 19 *Cyp* genes were consistent with paired FROZ (Supplementary Table 9). Comparison of preservation-related differences (FROZ *vs.* FFPE) across the 11 *Acot* and 43 *Cyp* family marker genes identified in FROZ samples by RNA-Seq demonstrated that matched FFPE samples tended to have fewer gene counts relative to paired FROZ, which was often significant. This pattern was evident for 9 of 11 *Acot* and 39 of 43 *Cyp* markers (paired Wilcoxon Signed Rank Test, FDR-adjusted p-value <0.05). The magnitude of these differences in RNA-Seq gene counts between FFPE and FROZ samples varied across markers but generally increased at higher DCA dose levels. For instance, *Acot* and *Cyp* counts from RNA-Seq FROZ samples tended to be 94.3 ± 8.3% and 96.4 ± 5.2% higher (median ± median absolute deviation) than RNA-Seq FFPE at 313 and 429 mg/kg-d, respectively. Analysis of percent changes for each marker gene for RNA-Seq FROZ *vs.* FFPE by dose level is available in Supplementary Table 10.

For TempO-Seq, we detected 9 *Acot* and 29 *Cyp* family genes with significant dose-dependent trends for FROZ samples (Kruskal-Wallis Test, p-value <0.05 and Jonckheere’s Trend Test, p-value <0.05). All these marker genes were also identified as significant in the FROZ RNA-Seq data set. TempO-Seq analysis of FFPE samples identified slightly fewer *Acot* (n = 6) and *Cyp* (n = 14) genes compared to paired TempO-Seq FROZ data, but all matched those identified in FROZ samples from either platform (Supplementary Table 11). The preservation-related changes (FROZ *vs.* FFPE) identified in TempO-Seq paired samples across *Acot* and *Cyp* genes were less drastic than those observed for RNA-Seq. For instance, 5 of 11 *Acot* and 33 of 43 *Cyp* had significant preservation-related differences between TempO-Seq FROZ and matched FFPE datasets (paired Wilcoxon Signed Rank Test, FDR-adjusted p-value <0.05). The changes did not seem to be influenced much by DCA dose level with TempO-Seq FROZ samples having between 28.3 ± 73–34.0 ± 76% greater counts (median ± median absolute deviation in percent change) compared to paired TempO-Seq FFPE (Supplementary Table 12).

**Fig. 7:**
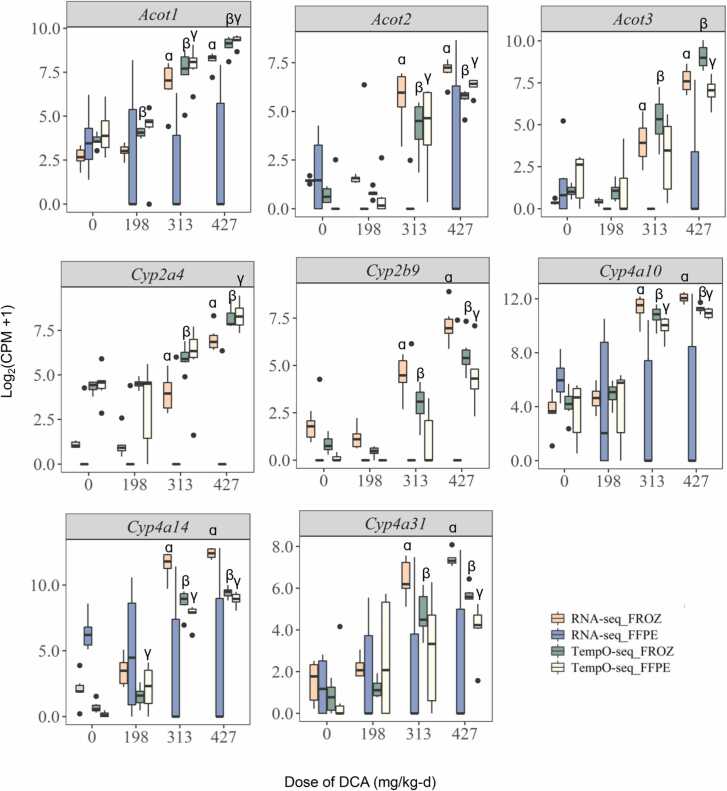
RNA-Seq FROZ, TempO-Seq FROZ and TempO-Seq FFPE detect similar top ranked DCA marker DEGs by fold change. Association in dose-dependent changes of DCA marker genes across preservations and platforms. CPM-normalized counts were offset by + 1 and then log_2_ transformed. ᵅ Designates significant difference between DCA dose level *vs* vehicle control for RNA-Seq FROZ. ᵝ Designates significant difference between DCA dose level *vs* vehicle control for TempO-Seq FROZ. ᵞ Designates significant difference between DCA dose level *vs* vehicle control for TempO-Seq FFPE. CPM-counts per million, DCA- dichloroacetic acid, FFPE-formalin-fixed paraffin-embedded, FROZ-frozen, RNA-Seq- RNA-Sequencing, FDR- false discovery rate. Significance determined by Wilcoxon Rank Sum Test, FDR-adjusted p-value < 0.05.

**Fig. 8:**
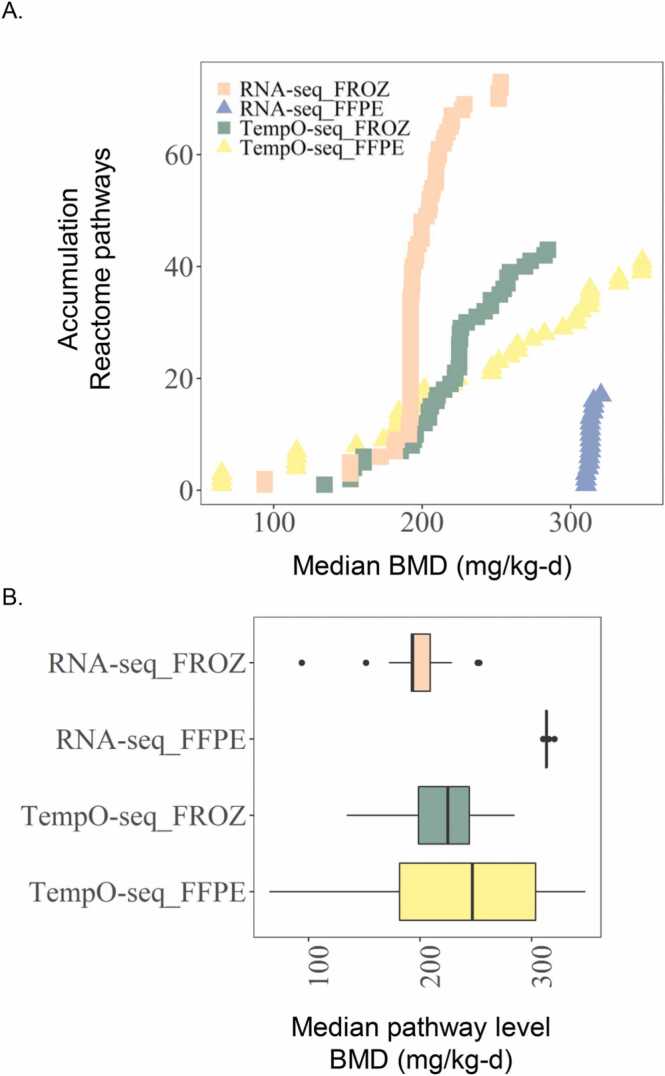
Gene set based BMD_T_ values identified for paired RNA-Seq FROZ and FFPE and paired TempO-Seq FROZ and FFPE liver tissue samples. A. Accumulation plots of lowest median BMD_T_ derived from mapping genes/probes to signaling pathways in Reactome. Enrichment based on at least 3 or more genes and a Fisher’s Exact Test, two-tailed, p-value < 0.05. B. Boxplots show distribution in pathway level BMD_T_ results across sample types and methods. Abbreviations: FFPE-formalin-fixed paraffin-embedded, FROZ-frozen, RNA-Seq- RNA-Sequencing, BMD_T_ - transcriptomic benchmark dose.

Focus on the top 10 DCA induced DEGs by rank in absolute fold change for FROZ across either platform identified several *Acot* and *Cyp* genes: *Acot1*, *Acot2*, *Acot3*, *Cyp2a4*, *Cyp2b9*, *Cyp4a10*, *Cyp4a14*, and *Cyp4a31*. RNA-Seq FROZ samples displayed significant increases in *Acot1*, *Acot2*, *Acot3*, *Cyp2a4*, *Cyp2b9*, *Cyp4a10*, *Cyp4a14*, and *Cyp4a31* at 313 and 427 mg/kg-d compared to vehicle control (Wilcoxon Rank Sum Test, FDR-adjusted p-value <0.01) (Fig. 6, Supplementary Table 13). Paired RNA-Seq FFPE samples showed a variable pattern of increased fold changes with increased dose compared to vehicle controls. These fold changes were less than those identified in paired RNA-Seq FROZ samples. None of the pairwise comparisons achieved significance in RNA-Seq FFPE samples even at the highest DCA dose level compared to vehicle control. For example, the highest DCA-induced gene in RNA-Seq FROZ samples, *Cyp4a14*, showed increased expression at all dose levels ranging from 2.6 to 1023.2-fold relative to vehicle control, whereas paired FFPE samples only showed increased expression that ranged from 3.3 to 15.5-fold (Fig. 7, Supplementary Table 13). Interestingly, when DCA significantly affected *Acot* and *Cyp* marker genes in RNA-Seq FFPE samples, down regulation was typically observed. This was frequently seen in the *Cyp2* family of genes (Supplementary Table 10). The downregulation mostly corresponded with similar DCA-related down regulation for the same marker genes in paired RNA-Seq FROZ but the magnitude in fold changes tended to be slightly higher in FFPE Fig. 8.

Similar to RNA-Seq FROZ samples, TempO-Seq analysis of FROZ samples demonstrated significant increases in DCA-induced gene expression for the top ranked *Acot* and *Cyp* DEGs at 313–427 mg/kg-d *vs.* vehicle controls (Wilcoxon Rank Sum Test, FDR-adjusted p-value <0.01). TempO-Seq also detected a significant increase in *Acot1* at 198 mg/kg-d *vs.* vehicle control in FROZ samples, which was not observed in the RNA-Seq dataset (Fig. 7, Supplementary Table 11). All TempO-Seq FFPE samples also identified significant induction in the top ranked *Acot* and *Cyp* DEGs at the highest dose level compared to vehicle control. *Acot1*, *Acot2*, *Cyp4a10* and *Cyp4a14* also showed significantly increased gene expression at 313 mg/kg-d (Wilcoxon Rank Sum Test, FDR-adjusted p-value <0.05) compared to vehicle control. The degree of gene expression changes across DCA dose levels was similar between FFPE and FROZ for TempO-Seq with FFPE sometimes demonstrating higher magnitude fold changes. As with RNA-Seq of FROZ samples, TempO-Seq identified *Cyp4a14* as having the highest rank in fold-change expression for both FFPE and FROZ samples. TempO-Seq detected a 1.9–414.7-fold increase in *Cyp4a14* across 198–427 mg/kg-d dose levels of DCA in FROZ samples while it detected a 6.5–447.0-fold increase in FFPE samples. The level of induction at 427 mg/kg-d was not quite as high that measured by RNA-Seq analysis of FROZ samples but still remarkably similar and significant in TempO-Seq analysis of both FROZ and FFPE samples (Fig. 7, Supplementary Table 13). TempO-Seq analysis of FFPE samples did not demonstrate the higher magnitude fold changes in marker genes significantly downregulated by DCA exposure that was observed in RNA-Seq of FFPE samples.

### 3.4 For aged FFPE samples, RNA-Seq had lower numbers of genes suitable for BMD modeling compared to TempO-Seq

BMD analysis was performed to determine how well individual genes and gene sets reflected dose-dependent changes in expression, pathways and gene ontologies (GO) across preservation types. After best model fit selections and mapping to enriched signaling pathways or gene sets, we determined a BMD_T_ of 93.9 mg/kg-d for FROZ RNA-Seq samples, which was 3.3-fold lower than the paired FFPE samples (BMD_T_ = 310.3 mg/kg-d) (Table 1). When genes with good model fits were mapped to GO biological processes, we identified a BMD_T_ for FROZ samples that was slightly higher than the pathway results at 122.3 mg/kg-d. This was not the case for FFPE which was similar to the pathway level BMD at 304.1 mg/kg-d (Supplementary Table 14); however, GO and pathway-based gene set mapping were still remarkably similar within each preservation group. The reduced sensitivity of RNA-Seq FFPEs samples (higher BMD_T_) was likely due to very few genes undergoing BMD_T_ modeling (21). However, enough of those 21 genes demonstrated good dose-dependent curve fits and mapped to sufficient pathways or GO biological processes (17 pathways or 59 GO sets) to identify a lowest median BMD_T_ for both instances. In contrast, 739 genes in RNA-Seq FROZ samples had good curve fits and mapped to 73 pathways or 438 GO sets demonstrating much better sensitivity (Supplementary Table 13). Mapping of FROZ and FFPE genes to pathways identified enrichment in *synthesis of bile acids and bile salts via 27-hydroxycholesterol* and *platelet degranulation*, whereas enriched GO terms were *acylglycerol homeostasis* and *reproductive process*, respectively.

**Table 1:** Lowest median BMD derived from signaling pathway analysis.

Group	Reactome Pathway Name	Lowest Median BMD_T_ (mg/kg-d)	Lowest Median BMDL_T_ (mg/kg-d)	Fisher's Exact Two-Tailed p-value	No. Genes in Lowest Enriched Pathway	Total enriched pathways	Ratio BMD_T_/BMD_A_
RNA-Seq FROZ	synthesis of bile acids and bile salts *via* 27-hydroxycholesterol	93.9	73.8	5.70E-03	3	73	2.1
RNA-Seq FFPE	platelet degranulation	310.3	200.7	2.00E-09	5	17	7.1
TempO-seq FROZ	initial triggering of complement	134.2	101.4	6.50E-03	3	43	3.1
TempO-seq FFPE	chylomicron remodeling	65	45.4	2.10E-04	3	41	1.5

Lowest median benchmark doses were selected by Fisher’s Exact Two Tail Test (p-value <0.05 and genes ≥3). BMD (BMDL) for liver adenoma or carcinoma 43.7 (12.8) mg/kg-d.

When focusing on the individual genes that made up the BMD_T_ curve fits, clear differences were evident for FFPE and FROZ RNA-Seq samples. Despite identifying a lowest median pathway level BMD_T_ that was relatively similar (within 3.3-fold) to paired FROZ, there were not many genes in common between FFPE and FROZ that passed the ANOVA test and underwent BMD modeling (6 of 21). The curve fits for all six commonly modeled genes in FFPE and paired FROZ showed similar downward regression trends but the best identified model tended to differ between the two preservation groups (see Supplementary Figure 3, for representative examples). Moreover, all 21 FFPE dose response modeled genes demonstrated downward linear trends.

BMD analysis of FFPE data showed better concordance with paired FROZ for TempO-Seq compared to RNA-Seq. The lowest median signaling pathway BMD_T_ identified in FROZ samples was 134.2 mg/kg-d, which was 2.1-fold higher than the corresponding FFPE samples (65.0 mg/kg-d) (Table 1). The lowest median enriched GO term BMD_T_ values for FROZ and FFPE samples at 93.3 and 81.7 mg/kg-d, respectively (Supplementary Table 12) and were quite similar to the pathway level results. This finding was likely due to the relatively high number of genes (493) undergoing BMD_T_ modeling for TempO-Seq FFPE samples, which was only 1.5-fold fewer than those modeled for matched FROZ (722 genes). Enrichment analysis of TempO-Seq FROZ and FFPE samples identified *initial triggering of complement* and *chylomicron remodeling,* respectively, as the lowest median pathways, while GO enrichment identified *positive regulation of protein deacetylation* and *positive regulation of histone modification,* respectively. The total number of enriched pathways and GO terms were similar for TempO-Seq datasets at 474 pathways or 3761 GO terms (FFPE) and 457 pathways or 3879 GO terms (FROZ) (Supplementary Table 15).

When comparing pathway level BMD_T_ results across sequencing methods, TempO-Seq FROZ and FFPE were similar to RNA-Seq FROZ results (TempO-Seq FROZ results were 1.4-fold higher and TempO-Seq FFPE results were 1.4-fold lower) (Fig. 8). However, the TempO-Seq FFPE results appear more variable compared to RNA-Seq FROZ and TempO-Seq FROZ especially with genes that were only modestly induced by DCA treatment. When comparing the lowest median pathway results from the 6-day DCA study to the traditional BMD_A_ from the chronic reference DCA study, data from RNA-Seq FROZ, TempO-Seq FROZ, and TempO-Seq FFPE samples identified gene-set based points of departure that were 2.1, 3.1, and 1.5-fold higher than the BMD for liver adenoma and carcinoma (43.7 mg/kg-d), respectively. The BMD_T_ results from RNA-Seq FFPE was 7.1-fold higher than the traditional BMD value for DCA (Table 1).

## 4 Discussion

In this study, we predicted that a targeted sequencing platform would result in enhanced detection of gene expression responses in aged (>20 year-old) FFPE tissue samples compared to a standard whole-genome RNA-Seq platform. TempO-Seq analysis of FFPE samples showed improved global sequencing metrics, detection of treatment-related counts and DEGs, and concordance with paired FROZ samples in fold-change values and BMD estimates compared to RNA-Seq FFPE samples. Post-alignment quality readouts further suggested better performance of TempO-Seq *vs.* RNA-Seq with aged FFPE samples. These differences may be due to the ability of the probe-based method to detect genes without expending reads on intronic and intergenic sequences, in contrast to conventional RNA-Sequencing of FFPE samples, thereby circumventing some of the fixation-related damage to nucleic acids. Moreover, BMD modeling estimates in archival FFPE samples suggested that gene responses detected in short term studies may provide a useful way to identify target pathways and estimate biologic potency in the absence of longer term studies.

Probe-based sequencing of archival FFPE RNA showed a marked improvement relative to total RNA-Seq. TempO-Seq of FFPE-derived RNA was able to detect more genes at lower expression levels than RNA-Seq of FFPE, indicating less impact of formaldehyde-induced RNA modifications. This result may be due, in part, to the lack of an RNA isolation step with TempO-Seq, which could lead to nucleic acid loss during purification and elution [34]. RNA loss during isolation may also explain why only the most abundant RNA transcripts were detected in the RNA-Seq of FFPE tissue samples, as many crosslinked nucleic acids and less expressed transcripts are eluted [34]. However, direct lysis of tissue samples can be problematic with TempO-Seq in that conventional methods of ascertaining RNA quality and abundance (*i.e*. Bioanalyzer, NanoDrop and Qubit) prior to sequencing are not available.

Another potential reason for the poor performance of RNA-Seq with very old FFPE samples could be the lack of alignment specificity. Many studies have shown that RNA-Seq of FFPE samples results in an increase in intronic read alignment at the expense of exonic reads compared to paired FROZ samples, including the present study [1], [14], [17], [18], [21], [37]. This shift seems to increase with sample age and time in formalin [18], [37] and therefore poses a particular challenge with older FFPE tissue samples. In a recent report by Jones et al. [20], the authors noted that formalin fixation may be disrupting post-transcriptional processing of pre-mRNA, leading to retention of intronic regions or preventing their digestion through proteins cross-linked to the nucleic acid [20]. Regardless, the exome specificity of TempO-Seq may help overcome this challenge by focusing sequencing on probes targeting exonic regions, thereby increasing sensitivity to detect changes in gene expression. Consistent with this idea, > 95% of reads aligned to exonic regions of the reference genome in our dataset. In addition, the small oligonucleotide size of the TempO-Seq detector probes likely avoids some of the molecular modifications that impeded probe binding and cDNA synthesis during library preparation of RNA derived from FFPE samples. For RNA-Seq of FFPE tissue samples, which is attempting to sequence the entire transcriptome rather short exon-targeted probes, variable fragmentation and formaldehyde adducts would likely interfere with cDNA synthesis [23], [34], [36] and potentially divert reads to areas not of interest (*i.e*. intergenic and intronic), negatively impacting differential gene expression analysis. Therefore, when the experimental goal is to measure changes in gene expression based on current gene knowledge and probes, TempO-Seq has an advantage over RNA-Seq with aged archival FFPE tissue samples but it may not necessarily be ideal for novel discovery applications with higher quality samples.

Gene expression data from TempO-Seq of FFPE samples was successfully used for dose response modeling of gene expression. With the need for more rapid assessment of the large number of chemicals in commerce, there is an increasing focus on incorporating NAMs into chemical risk assessment. While the ultimate goal of NAMs is to use non-animal approaches in risk assessment, the Lautenberg Act implies that any approach to reduce, refine, or replace animal use is a NAM. The current manuscript provides evidence that short term *in vivo* studies coupled with transcriptomics-based BMD analysis can be a useful NAM in determining the dose at which relevant biological or pathway-level effects occur. Here, we identified BMD_T_ estimates following a short term exposure to DCA as a reference toxicant. Across both sequencing platforms, the 6-day BMD_T_ values were within 7.1-fold of the 2-year cancer BMD_A_ for hepatocellular carcinoma or adenoma (mouse BMD_A_ = 43.7 mg/kg-d, [8]). This finding is consistent with other studies testing a variety of environmental chemicals, which have shown that the lowest median pathway BMD_T_ and BMD_A_ are within 2–3-fold of the chronic cancer and noncancer BMD_A_s when focusing on the target tissue [33] or 10-fold when focusing on sentinel tissues of effect like liver or kidney [16], [19]. However, the higher sensitivity of TempO-Seq gene detection in old FFPE tissue samples resulted in BMD_T_ values for gene sets that were more consistent with matched FROZ and RNA-Seq FROZ and more closely aligned with the BMD_A_.

DCA is a known rodent liver tumorigen and inhibits pyruvate dehydrogenase kinase, which increases pyruvate dehydrogenase activity leading to glycogen accumulation in the liver, lipid effects, and metabolic disruption [8]. The lowest enriched gene sets for TempO-Seq FFPE samples were quite consistent with known or suspected DCA effects [38], [8]. Moreover, DCA dependent DEGs and marker genes identified by TempO-Seq of FFPE samples, demonstrated more consistent dose-dependent changes in gene expression compared to RNA-seq of FFPE resulting in BMD_T_ calculations quite similar to FROZ samples across both platforms (RNA-Seq and TempO-Seq FROZ). While RNA-Seq of FFPE samples detected some dose-dependent trends in CPM-normalized DCA marker genes, no *Cyp* or *Acot* family genes were included in BMD_T_ modeling results. All were removed by the standard statistical filters.

## 5 Conclusions

Probe-based TempO-Seq offers a promising tool for obtaining gene expression data from older FFPE samples, in support of chemical mode of action, dose response analysis, and biomarker discovery. While limited to a single case study, this particular analysis had several unique features including a gold standard method (RNA-Seq), referent sample type (FROZ), and well-studied chemical (DCA). Targeted sequencing clearly outperformed standard whole-genome RNA-Seq in terms of sensitivity and dose response estimates for our aged FFPE liver tissue samples. While TempO-Seq is limited by a set number of genes, it is exome-targeted and does not require RNA isolation or cDNA synthesis, both of which may limit the successful detection of differentially expressed genes in conventional whole genome sequencing of very old FFPE tissue samples. Some additional potential limitations of performing gene expression analysis on aged archival tissue samples in general include the potential for identifying false positives and difficulty in detecting modest experimental effects. Modest gene expression changes from archival samples should only be excepted as significant with corollary evidence. However, analysis of multi-dose-level experiments, like the one presented, here can increase confidence in identifying truly significant genes by focusing on genes with changes across several dose levels and by focusing on those genes with similar dose trends. Changes in related transcripts (*e.g. Cyp* gene clusters) can also provide corroborating evidence in identifying true significant differences in aged archival samples. Overall, this work has implications for advancing the use of NAMs in risk assessment as part of retrospective investigations, in which archival samples may be used to obtain new molecular data without the need for *de novo* studies.

## Disclaimer

The research described in this article has been reviewed by the U.S. EPA and approved for publication. Approval does not signify that the contents necessarily reflect the views or the policies of the Agency. Mention of trade names or commercial products does not constitute endorsement or recommendation for use.

## Declaration of Competing Interest

The authors declare that they have no known competing financial interests or personal relationships that could have appeared to influence the work reported in this paper.

## Appendix A Supplementary material

Supplementary material.

Supplementary Fig. 1 Comparison of log_2_ fold change induced by DCA exposure in FROZ DEGs *vs.* any matched gene in paired FFPE liver tissue samples using the TempO-Seq platform. Regression analysis of the intersection in log_2_ transformed fold-change values induced by DCA at A. 427 mg/kg-d *vs.* vehicle control, B. 313 mg/kg-d *vs.* vehicle control, and C. 198 mg/kg-d *vs.* vehicle control D. Total DCA-induced DEGs from TempO-Seq FROZ and all genes from paired FFPE liver tissue samples as well as overlap relative to FROZ samples. DEGs were defined with these parameters: absolute value (fold change)> 2, FDR-adjusted p-value< 0.05. Abbreviations. DEG-differentially expressed genes, DCA- dichloroacetic acid, FFPE-formalin-fixed paraffin-embedded, FROZ-frozen, RNA-Seq- RNA-Sequencing, FDR- false discovery rate.

.

Supplementary Fig. 2 Comparison of log2 fold change induced by DCA exposure in FROZ DEGs identified by RNA-Seq *vs.* any matched gene in FFPE liver tissue samples identified by TempO-Seq. Regression analysis of the intersection in log_2_ transformed fold-change values at A. 427 mg/kg-d *vs.* vehicle control, B. 313 mg/kg-d *vs.* vehicle control, and C. 198 mg/kg-d *vs.* vehicle control D. Total DCA-induced DEGs from RNA-Seq FROZ and all genes from FFPE liver tissue samples identified by TempO-Seq as well as overlap relative to RNA-Seq FROZ samples. DEGs were defined with these parameters: absolute value (fold change)> 2, FDR-adjusted p-value< 0.05. Abbreviations. DEG-differentially expressed genes, DCA- dichloroacetic acid, FFPE-formalin-fixed paraffin-embedded, FROZ-frozen, RNA-Seq- RNA-Sequencing, FDR- false discovery rate.

.

Supplementary Fig. 3 Investigation of best model curve fits for BMD analysis of select representative genes from RNA-Seq FFPE that demonstrated a significant DCA *vs.* vehicle control effect that also were identified in RNA-Seq FROZ (significance by ANOVA, p-value <0.05, maximum fold change >2). Parameters for best model fit selection described in Methods. ANOVA-analysis of variance, DCA- dichloroacetic acid, FFPE-formalin-fixed paraffin-embedded, FROZ-frozen, seq-sequencing, BMD-benchmark dose.
