# Supplementary figures and images for: Case study: Targeted RNA-sequencing of aged formalin-fixed paraffin-embedded samples for understanding chemical mode of action
Source: Toxicol Rep. 2022 Apr 18;9:883–94. doi: 10.1016/j.toxrep.2022.04.012 (PMC9742836; doi:10.1016/j.toxrep.2022.04.012)

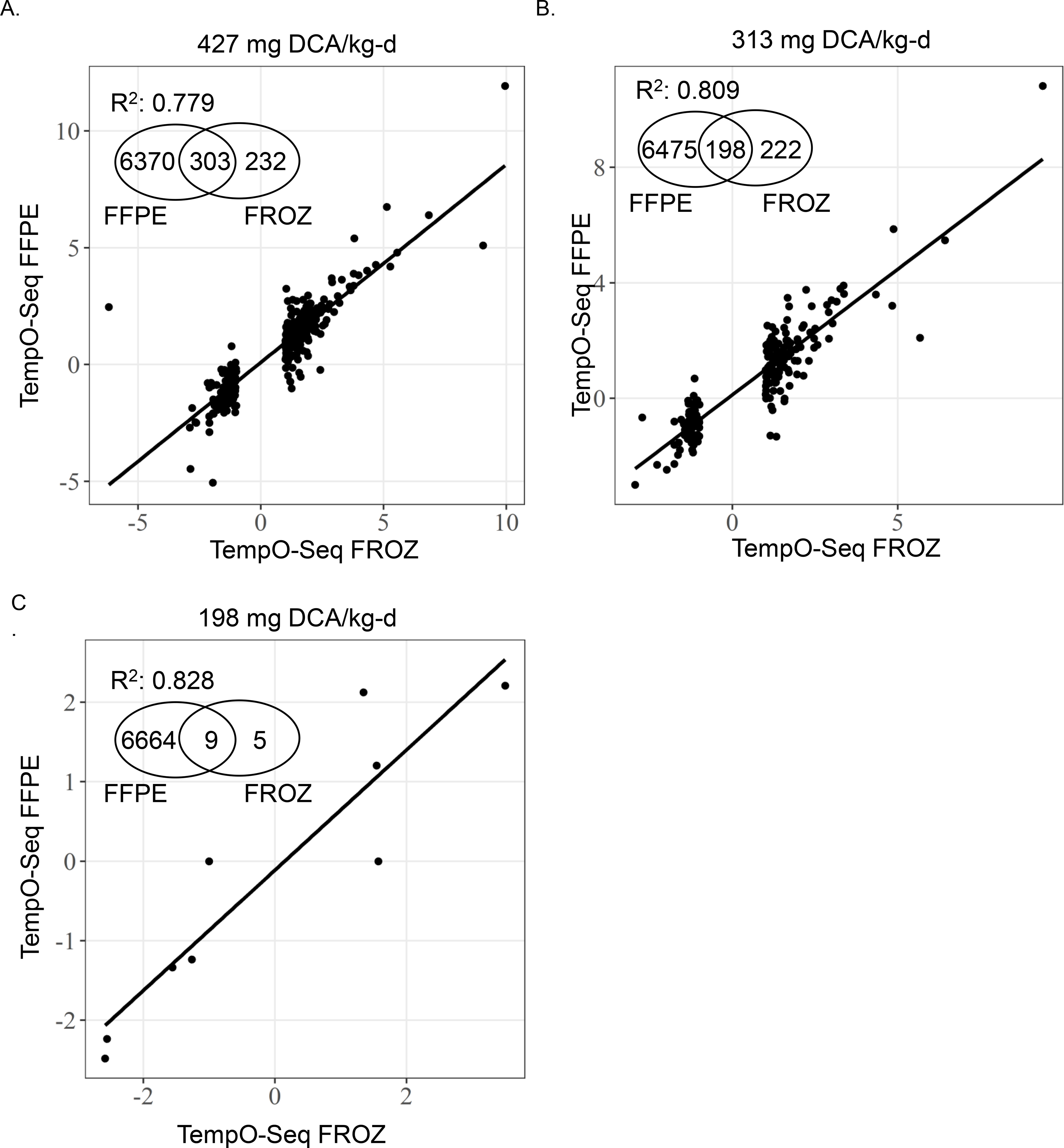

Supplement: Supplementary Fig. 1 — Comparison of log2 fold change induced by DCA exposure in FROZ DEGs vs. any matched gene in paired FFPE liver tissue samples using the TempO-Seq platform. Regression analysis of the intersection in log2 transformed fold-change values induced by DCA at A. 427 mg/kg-d vs. vehicle control, B. 313 mg/kg-d vs. vehicle control, and C. 198 mg/kg-d vs. vehicle control D. Total DCA-induced DEGs from TempO-Seq FROZ and all genes from paired FFPE liver tissue samples as well as overlap relative to FROZ samples. DEGs were defined with these parameters: absolute value (fold change)> 2, FDR-adjusted p-value< 0.05. Abbreviations. DEG-differentially expressed genes, DCA- dichloroacetic acid, FFPE-formalin-fixed paraffin-embedded, FROZ-frozen, RNA-Seq- RNA-Sequencing, FDR- false discovery rate. [file mmc2.jpg]

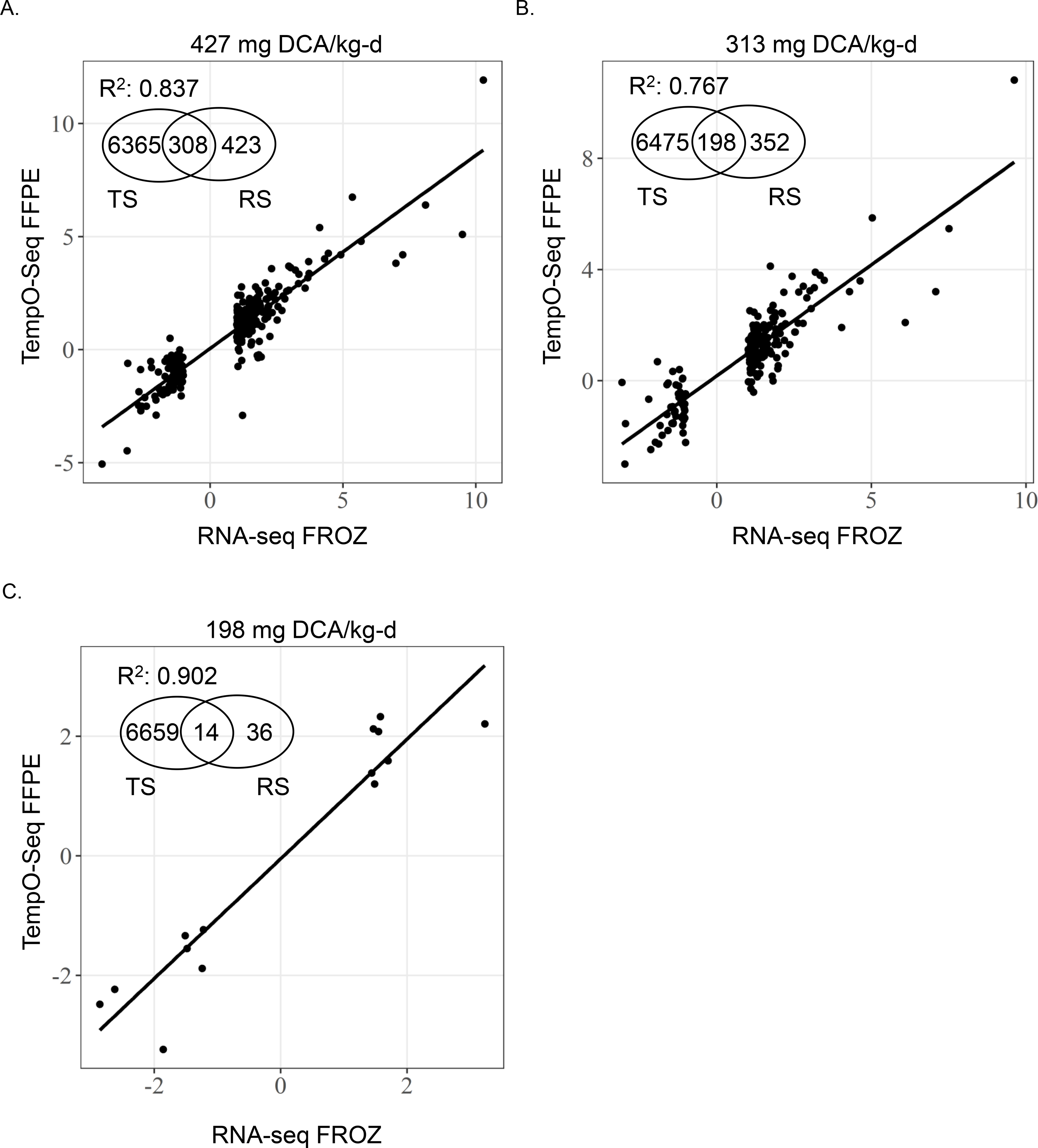

Supplement: Supplementary Fig. 2 — Comparison of log2 fold change induced by DCA exposure in FROZ DEGs identified by RNA-Seq vs. any matched gene in FFPE liver tissue samples identified by TempO-Seq. Regression analysis of the intersection in log2 transformed fold-change values at A. 427 mg/kg-d vs. vehicle control, B. 313 mg/kg-d vs. vehicle control, and C. 198 mg/kg-d vs. vehicle control D. Total DCA-induced DEGs from RNA-Seq FROZ and all genes from FFPE liver tissue samples identified by TempO-Seq as well as overlap relative to RNA-Seq FROZ samples. DEGs were defined with these parameters: absolute value (fold change)> 2, FDR-adjusted p-value< 0.05. Abbreviations. DEG-differentially expressed genes, DCA- dichloroacetic acid, FFPE-formalin-fixed paraffin-embedded, FROZ-frozen, RNA-Seq- RNA-Sequencing, FDR- false discovery rate. [file mmc3.jpg]

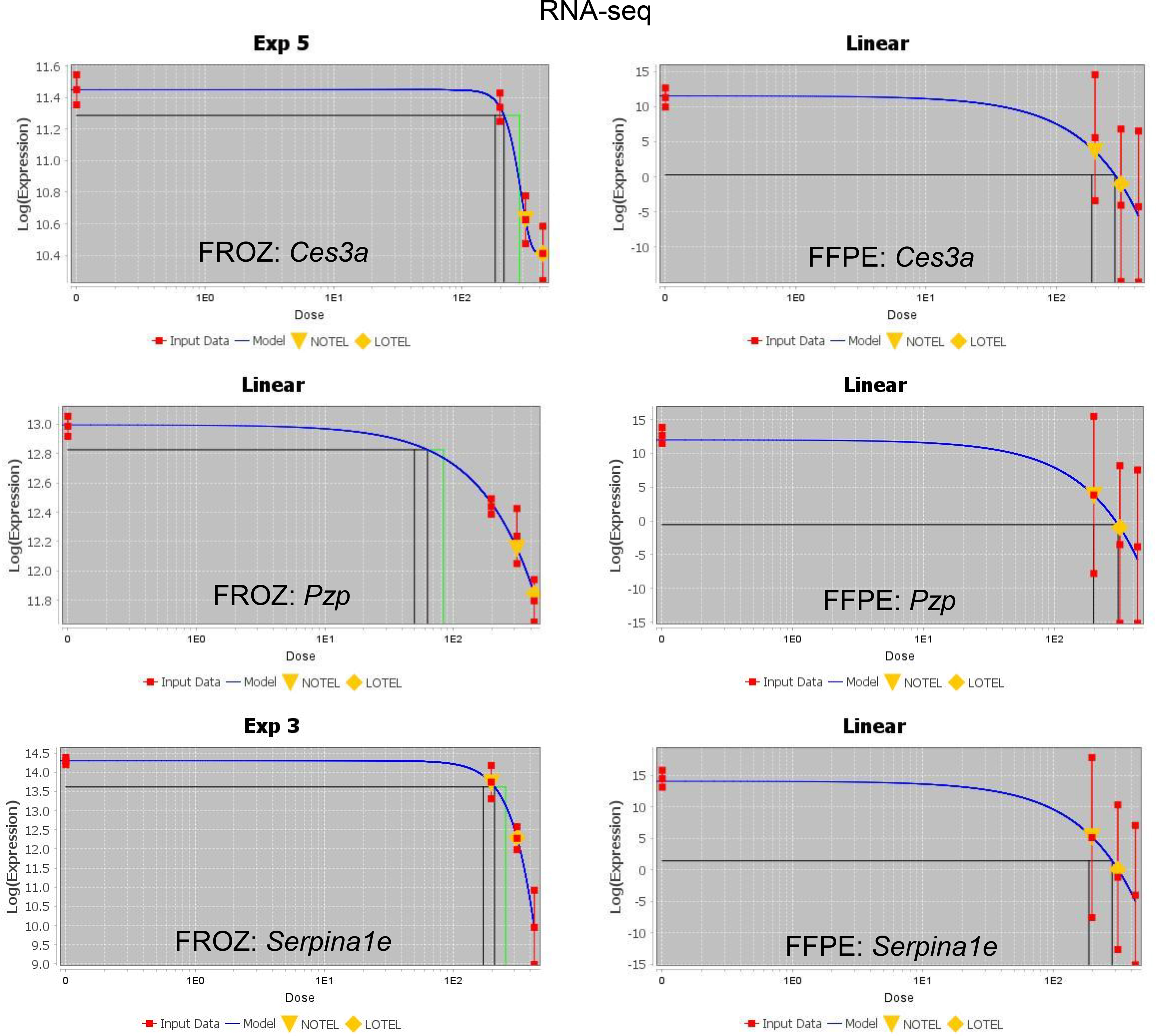

Supplement: Supplementary Fig. 3 — Investigation of best model curve fits for BMD analysis of select representative genes from RNA-Seq FFPE that demonstrated a significant DCA vs. vehicle control effect that also were identified in RNA-Seq FROZ (significance by ANOVA, p-value <0.05, maximum fold change >2). Parameters for best model fit selection described in Methods. ANOVA-analysis of variance, DCA- dichloroacetic acid, FFPE-formalin-fixed paraffin-embedded, FROZ-frozen, seq-sequencing, BMD-benchmark dose. [file mmc4.jpg]
